# Gut Resistome and Hearing Loss in Young Adults: A Preliminary Study on the Interplay Between Microbial Resistance and Auditory Health

**DOI:** 10.3390/antibiotics14121241

**Published:** 2025-12-08

**Authors:** Julia Almazán-Catalán, Paula Carpizo-Zaragoza, Diana Penalba-Iglesias, María Luisa Sánchez, Daniel González-Reguero, Sara Bueno, Marina Robas-Mora, Gregorio Varela-Moreiras, Teresa Partearroyo, Pedro Jiménez-Gómez

**Affiliations:** 1 Grupo USP-CEU de Excelencia “Nutrición para la Vida (Nutrition for Life)”, Ref: E02/0720, Department of Pharmaceutical and Health Sciences, Faculty of Pharmacy, San Pablo University, CEU Universities, Urbanización Monteprincipe, 28660 Boadilla del Monte, Spain; julia.almazancatalan@ceu.es (J.A.-C.); paula.carpizozaragoza@usp.ceu.es (P.C.-Z.); gvarela@ceu.es (G.V.-M.); t.partearroyo@ceu.es (T.P.); 2 Department of Pharmaceutical and Health Sciences, San Pablo University, CEU Universities, Urbanización Montepríncipe, 28660 Boadilla del Monte, Spain; diana.penalbaiglesias@ceu.es (D.P.-I.); daniel.gonzalezreguero@ceu.es (D.G.-R.); marina.robasmora@ceu.es (M.R.-M.); 3 Department of Applied Chemistry and Biochemistry, Faculty of Pharmacy, San Pablo University, CEU Universities, Urbanización Montepríncipe, 28660 Boadilla del Monte, Spain; sanrodml@ceu.es (M.L.S.); buenofdz@ceu.es (S.B.); 4 Instituto Universitario CEU Alimentación y Sociedad, Faculty of Pharmacy, San Pablo University, CEU Universities, Urbanización Monteprincipe, 28660 Boadilla del Monte, Spain

**Keywords:** hearing loss, microbiome, resistome, microbial diversity

## Abstract

**Background**: Hearing loss (HL) affects more than 1.5 billion people worldwide and represents a major global health concern. Recent evidence suggests that alterations in gut microbial composition and antimicrobial resistance (AMR) may be linked to inflammatory and metabolic pathways that could influence auditory physiology. **Objectives:** This study aimed to explore the relationship between auditory function and the antimicrobial resistance in the gut microbiome of young adults. **Methods:** Fecal and auditory data were collected from young adults. Auditory function was assessed through pure-tone audiometry, and participants were classified according to the presence or absence of HL based on the American Speech-Language-Hearing Association (ASHA) criteria. Bacterial resistance was analyzed under aerobic and anaerobic conditions using disk diffusion and E-test methods to determine minimum inhibitory concentrations (MICs) for a panel of antibiotics. Gut microbiota composition was further characterized using quantitative polymerase chain reaction (qPCR) to quantify 15 key microbial taxa. **Results:** Overall, 40.9% of participants presented some degree of HL, with mild or slight HL being more frequent in women (53.3%) than in men (14.3%). Participants with HL exhibited significantly higher MICs for nalidixic acid, amoxicillin, and ciprofloxacin, as well as trends toward increased MIC variability for several other agents. Principal component analysis demonstrated distinct clustering of individuals without HL and greater dispersion among those with HL, suggesting higher interindividual variability in resistance profiles. These findings suggest potential associations between antimicrobial resistance and auditory function, possibly mediated through gut microbiome alterations. qPCR analyses demonstrated that *Faecalibacterium prausnitzii* abundance was significantly higher in individuals with HL and in those exhibiting greater resistance to amoxicillin. **Conclusions:** These findings provide preliminary evidence connecting the gut resistome with auditory function, supporting the emerging concept of a gut–ear–brain axis and underscoring the need for further research into microbiome-related mechanisms underlying HL.

## 1. Introduction

Hearing loss (HL) affects more than 1.5 billion people worldwide and represents a major global health problem due to its profound social, economic, and healthcare implications [1]. Its prevalence is rising globally, particularly among women and individuals aged 15 to 49 years, reflecting a concerning trend in younger populations [2]. This increase is largely attributed to recreational and occupational noise exposure, including attendance at loud music events and the use of personal listening devices (PLDs) or other unsafe listening practices [3,4,5]. The World Health Organization (WHO) recognizes recreational noise exposure as one of the leading preventable causes of sensorineural HL in young people, contributing to noise-induced HL. It is estimated that more than one billion individuals aged 12–35 years are exposed to potentially harmful sound levels through PLD use, posing serious risks to both physical and mental health [6].

The etiology of sensorineural HL is multifactorial, involving factors such as age, genetic predisposition, acute or chronic noise exposure, use of ototoxic drugs, and degenerative processes associated with chronic diseases [7,8,9]. Beyond these classical factors, recent evidence suggests that systemic elements, particularly the intestinal microbiome, may also influence auditory function [10]. Studies in animal models and preliminary human research indicate that the gut microbiome can modulate neuronal and sensory processes via immune regulation, metabolic signaling, and inflammatory pathways. In this context, it has been proposed that the effects of noise and other environmental factors on hearing may be mediated by complex systemic interactions, including a bidirectional relationship between the gut microbiome and the nervous system [10,11,12,13]. Within this framework, the concept of the gut–ear–brain axis has been proposed, suggesting that interactions among the microbiota, auditory system, and brain could influence the onset and progression of hearing loss, underscoring the importance of integrating dietary, environmental, and auditory factors [13,14].

In addition to this systemic framework, antimicrobial resistance (AMR) represents another pressing concern, driven by the excessive or inappropriate use of antibiotics [15]. AMR currently accounts for at least 700,000 deaths annually worldwide, a number projected to increase to nearly 10 million by 2050 in the absence of effective containment strategies [16]. Beyond increased mortality, AMR contributes to higher patient morbidity, greater healthcare costs, and the need to use more potent or broader-spectrum antibiotics [17], some of which have potential ototoxic effects, such as aminoglycosides [18] and certain non-aminoglycoside antimicrobials [19], thereby increasing the risk of drug-induced hearing impairment. In this context, national initiatives such as the Spanish National Plan Against Antibiotic Resistance (PRAN) 2025–2027 emphasize the urgent need for coordinated strategies to reduce antibiotic misuse, strengthen surveillance of resistance determinants—including within the human microbiome—and promote a One Health approach that links human, animal, and environmental health [20].

Despite these concerns, direct evidence linking AMR and HL is currently lacking. Investigating the intestinal resistome (the collection of antimicrobial resistance genes present in the gut microbiome) may help elucidate potential interactions among the microbiome, systemic inflammation and auditory function. Alterations in the composition and functionality of the gut microbiome have been associated with metabolic and immune processes that could indirectly influence the physiology of the auditory system [21,22]. Although the role of the gut microbiome in systemic and neurological functions is increasingly recognized, no studies to date have specifically examined the gut resistome as a potential factor influencing auditory health, representing a critical research gap.

Within this context, the present study aims to evaluate the potential relationship between auditory function and the presence of antimicrobial resistance genes in the gut microbiome of young adults. This exploratory approach seeks to generate preliminary evidence supporting the hypothesis that microbial resistance patterns may be linked to auditory outcomes, thereby contributing to a better understanding of the gut–microbiome–ear axis and its implications for hearing function.

## 2. Results

The final study sample consisted of 22 healthy volunteers, of whom 15 were women and 7 men. The median (interquartile range) age was 22 (20–24) years for women and 22 (21–23) years for men.

### 2.1. Hearing Evaluation of the Sample

To determine the degree of HL, the classification system of the American Speech–Language–Hearing Association (ASHA) was used as a reference [23].

**Figure 1:**
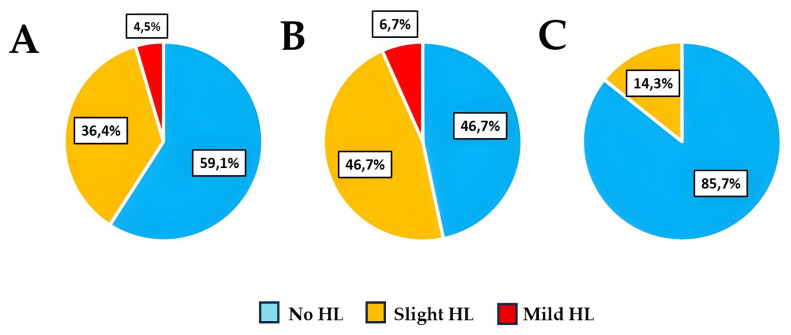
Air conduction audiometry results in the overall sample (**A**), in women (**B**), and in men (**C**). No HL: no hearing loss; Slight HL: slight hearing loss; Mild HL: mild hearing loss.

In the air conduction audiometry results, 59.1% of participants exhibited normal hearing, 36.4% showed slight HL, and 4.5% mild HL (Figure 1A). When stratified by sex, we found that 46.7% of women had normal hearing, while 53.3% exhibited some degree of HL, distributed as 46.7% slight HL and 6.7% mild HL (Figure 1B). In contrast, 85.7% of men had no hearing impairment, and only 14.3% presented slight HL (Figure 1C).

Despite the apparent differences between sexes, no statistically significant differences were observed in the air conduction audiometry results. Participants were therefore classified into two main groups based on hearing status (Table 1), with those with no hearing loss (“No HL”) and those with any degree of hearing loss (“Any degree of HL”), which included all participants with slight or mild impairment. No participants presented moderate or greater degrees of HL.

### 2.2. Analyisis of the Gut Microbiome

The analysis of antibiotic resistance profiles using the cenoantibiogram technique allowed the determination of minimum inhibitory concentrations (MICs) for each fecal sample under aerobic and anaerobic conditions. These data were grouped into components based on their similar behavior. Subsequently, a principal component analysis (PCA) was performed to identify patterns and trends in microbiome behavior at the population level, considering the presence or absence of any degree of HL.

#### 2.2.1. Comparative Analysis of Antibiotic Response

**Figure 2:**
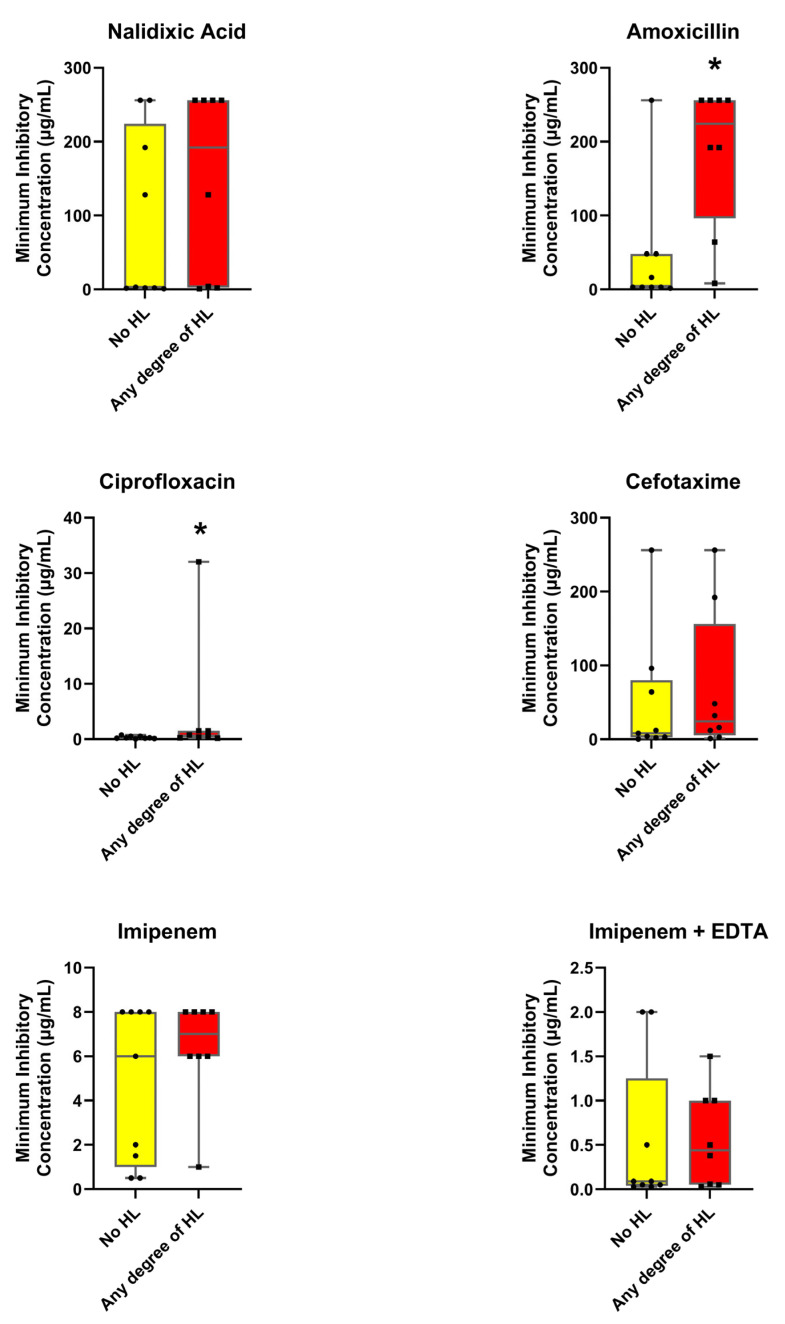
**Comparison of minimum inhibitory concentrations of antibiotics under aerobic conditions between patients without hearing loss and those with some degree of hearing loss.** Values are presented as median and interquartile range using box-and-whisker plots. * *p* ≤ 0.05 indicates significant differences between groups (Mann–Whitney U-test).

**Figure 3:**
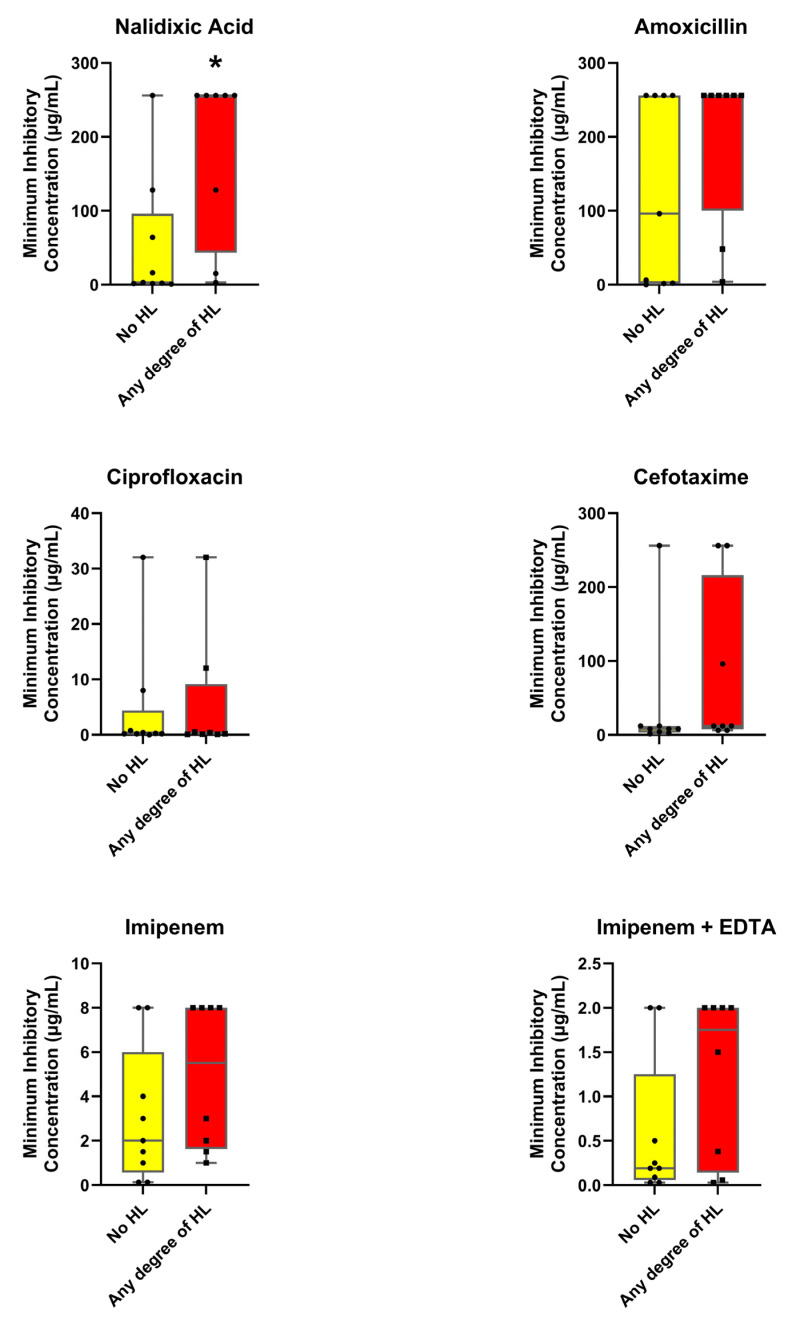
**Comparison of minimum inhibitory concentrations of antibiotics under anaerobic conditions between patients without hearing loss and those with some degree of hearing loss**. Values are presented as median and interquartile range using box-and-whisker plots. * *p* ≤ 0.05 indicates significant differences between groups (Mann–Whitney U-test).

The MIC values for the antibiotic’s amoxicillin, cefotaxime, imipenem, imipenem + EDTA, nalidixic acid, and ciprofloxacin were obtained using the cenoantibiogram technique under aerobic and anaerobic conditions, evaluating the influences of the HL state. The results (Figure 2 and Figure 3) revealed distinctive patterns between the groups.

As shown in Figure 2 and Figure 3, MIC values were generally higher in fecal samples from participants with HL compared to those without this condition. The median MIC was consistently elevated in isolates from the HL group, suggesting a general trend toward increased resistance. Significant differences between participants with and without HL were observed for several antibiotics. Under anaerobic conditions, nalidixic acid showed markedly higher median MIC values in the HL group (256 µg/mL [43.25–256.0]) compared with the group without HL (3.0 µg/mL [1.5–96.0]; *p* = 0.021). Under aerobic conditions, amoxicillin (224 µg/mL [96.0–256.0] vs. 3.0 µg/mL [3.0–48.0]; *p* = 0.008) and ciprofloxacin (0.625 µg/mL [0.2825–1.50] vs. 0.25 µg/mL [0.16–0.50]; *p* = 0.046) also exhibited significantly higher MICs among participants with HL. Although the remaining antibiotics did not reach statistical significance, their median values and dispersion tended to be greater in the HL group, suggesting a consistent pattern of elevated resistance across most agents tested.

#### 2.2.2. Principal Component Analysis (PCA)

**Figure 4:**
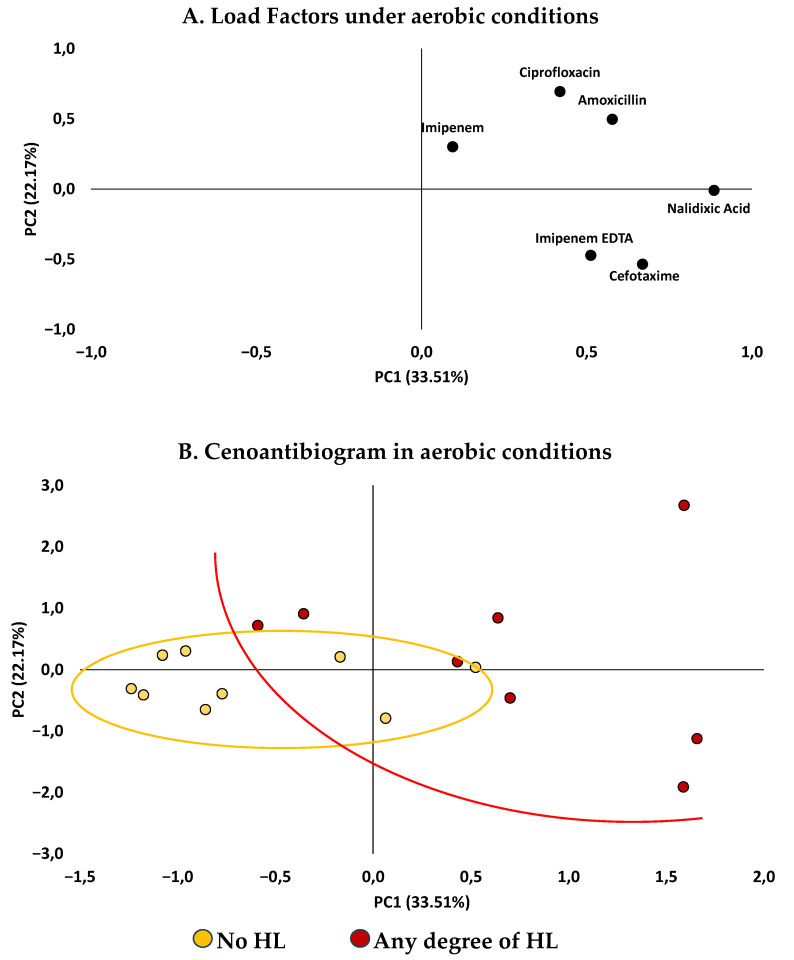
**Antibiotic response under aerobic conditions according to hearing loss status.** (**A**) Factor loading plot showing Component 1 (22.17%) and Component 2 (33.51%). (**B**) PCA biplot displaying the distribution of participants classified as “No hearing loss” and “Any degree of hearing loss.” Ellipses represent 95% confidence intervals for each group, illustrating partial clustering according to hearing status.

PCA was performed to explore the overall structure of antibiotic susceptibility patterns and its relationship between participants with and without HL. The first two principal components explained 55.67% of the total variance, with Component 1 explaining 22.17% of the variance and Component 2 explaining 33.51%. Under aerobic conditions (Figure 4), the scatter plot of the factor scores showed partial segregation between the groups. Participants with HL tended to cluster in the upper right quadrant, corresponding with high loadings on Component 1, which was mainly associated with ciprofloxacin, amoxicillin, and nalidixic acid. This distribution aligns with individual comparisons, reflecting distinct resistance profiles between groups.

**Figure 5:**
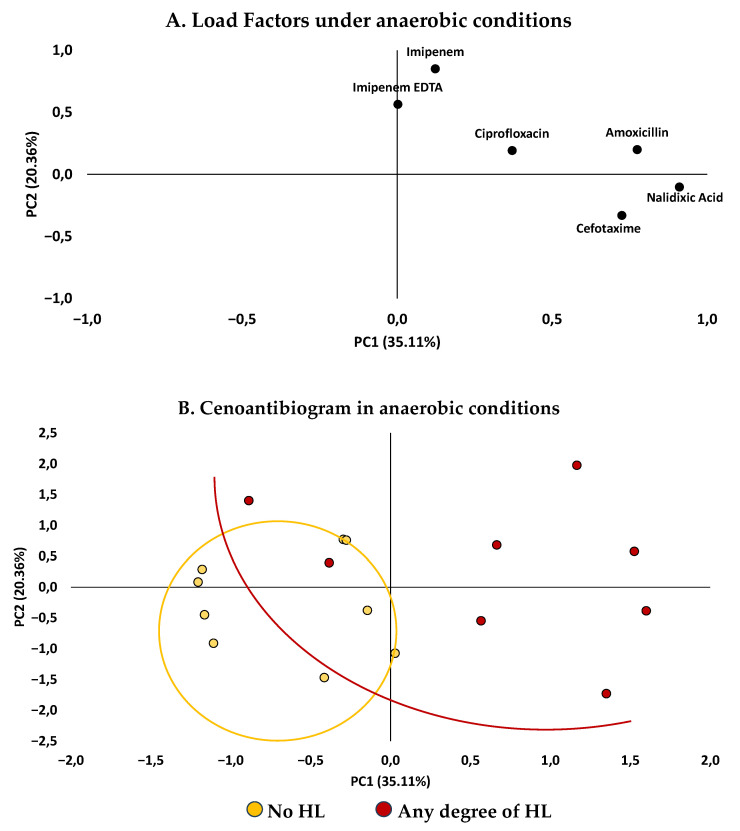
**Antibiotic response under anaerobic conditions according to hearing loss status.** (**A**) Factor loading plot showing Component 1 (22.17%) and Component 2 (33.51%). (**B**) PCA biplot displaying the distribution of participants classified as “No hearing loss” and “Any degree of hearing loss.” Ellipses represent 95% confidence intervals for each group, indicating a clearer separation of data points under anaerobic conditions.

Under anaerobic conditions (Figure 5), the separation between groups was even more evident. Component 2 emerged as the main discriminating axis, contrasting susceptibility to imipenem and imipenem + EDTA (positive loadings) with susceptibility to cefotaxime (negative loadings). Participants with hearing loss were positioned toward higher Component 2 scores, while those without hearing loss clustered in lower-score regions. The overall distribution suggests distinct multivariate patterns of antibiotic response according to hearing status.

#### 2.2.3. Relative Abundances

**Figure 6:**
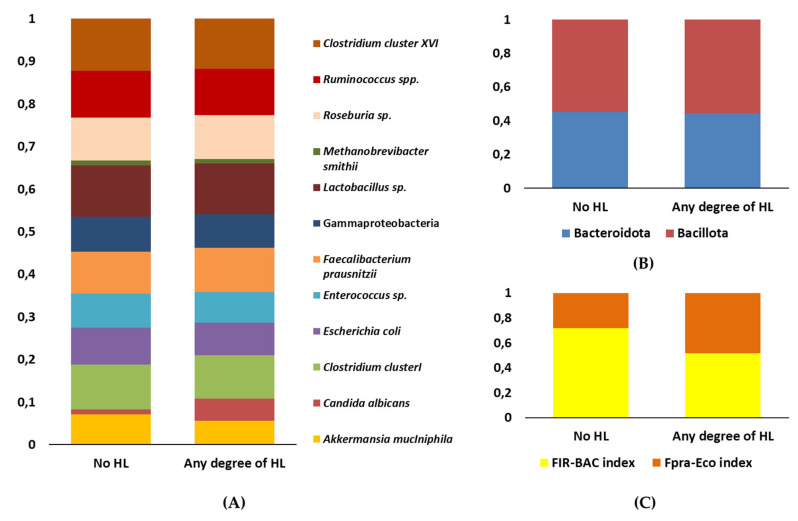
**Gut microbiota composition and functional indices according to HL status. Antibiotic response under anaerobic conditions according to hearing loss status.** The *Y*-axis represents relative abundance (**A**,**B**) or index value (**C**), while the *X*-axis distinguishes individuals without HL (No HL) from those with any degree of HL. Colors indicate bacterial taxa or phyla, as detailed in the legend. (**A**) Comparison of relative abundances at the taxon level, including genera, species, and clusters. (**B**) Comparison of the proportions of Bacteroidota and Bacillota phyla. (**C**) Comparison of Fpra-Eco and FIR-BAC indices, reflecting functional profiles of the gut microbiota.

To assess gut microbiota composition, total DNA was extracted from fecal samples using the NucleoSpin™ Soil DNA Kit (Macherey-Nagel GmbH & Co., Düren, Germany) according to the manufacturer’s instructions and eluted in a final volume of 100 µL. The relative abundances of 15 microbial markers representing the main phyla, groups, and genera of the gut microbiota were quantified using real-time quantitative polymerase chain reaction (qPCR). The markers analyzed included *Akkermansia muciniphila*, Bacteroidota, *Candida albicans*, Clostridium cluster I, *Escherichia coli*, *Enterococcus* sp., *Faecalibacterium prausnitzii*, Bacillota (formerly Firmicutes), Gammaproteobacteria, *Lactobacillus* sp., *Methanobrevibacter smithii*, *Roseburia* sp., *Ruminococcus* spp., Clostridium cluster XIV, and Eubacteria. Relative abundances were then compared between individuals without HL (No HL) and those with any degree of HL (Any degree of HL). The results of these analyses are presented in Figure 6.

Microbial composition analyses were performed in individuals without HL (No HL) and those with varying degrees of HL (Any degree of HL), with no notable differences observed in the overall gut microbiota composition between the groups. However, the relative abundance of *Faecalibacterium prausnitzii* was significantly higher in the HL group compared to No HL (Mann–Whitney U test, *p* = 0.010), suggesting a potential adaptive shift in the gut microbiota associated with auditory status. The Fpra-Eco index was significantly higher in the HL group, indicating an increased functional contribution of *F. prausnitzii* within the gut microbiota, whereas the FIR-BAC index did not show significant differences between groups.

To further investigate the relationship between gut microbiota composition and antibiotic resistance, analyses were focused on the antibiotics that had previously shown significant differences between HL and No HL groups. Individuals were stratified into two groups based on the median to distinguish high versus low resistance. Under these conditions, a significantly higher abundance of *Faecalibacterium prausnitzii* was observed in individuals with greater resistance to amoxicillin (Mann–Whitney U test, *p* = 0.008). In contrast, no significant differences in microbial abundances were detected when comparing groups based on resistance to ciprofloxacin and nalidixic acid.

## 3. Discussion

HL and AMR represent two major and expanding public health concerns worldwide, both associated with substantial impacts on quality of life and healthcare systems [1,24]. Although the present study is small and exploratory, the findings provide preliminary insights into potential links between auditory function and the intestinal resistome, which warrant further investigation.

The WHO has emphasized the growing vulnerability of young population to hearing impairment caused by recreational noise exposure, particularly through the widespread use of PLDs and attendance at loud music events [5]. Improper use of these technologies and chronic exposure to high sound levels have been recognized as preventable causes of early auditory dysfunction. In parallel, antibiotic resistance has emerged as a global health crisis, extending beyond human medicine to animal and environmental domains, and posing a significant threat to the effectiveness of current antimicrobial therapies [25].

Within this framework, the study of the intestinal resistome, the collection of antimicrobial resistance genes in the gut microbiome, has gained increasing importance as a functional biomarker of antibiotic exposure and broader lifestyle influences on microbial ecology. Alterations in the resistome may not only reflect prior antibiotic use but also mirror systemic processes, including immune and metabolic pathways, that could potentially influence extraintestinal functions [26,27,28].

Despite extensive research on each of these issues independently, no previous study has jointly examined the potential link between hearing loss and the intestinal resistome. Existing literature has largely focused on the direct ototoxicity of specific antibiotics, such as aminoglycosides [29] or macrolides [30], without exploring systemic or microbiome-mediated mechanisms that might contribute to auditory vulnerability. In this context, the present preliminary and exploratory study represents a pioneering approach to investigating the relationship between gut resistome profiles and auditory function in young adults, thereby contributing to the understanding of potential microbiome-mediated influences on hearing health.

### 3.1. Prevalence of Hearing Loss

In our cohort of 22 participants, 40.9% presented some degree of HL according to the ASHA classification [23]. When stratified by sex, 53.3% of women exhibited mild or slight HL compared to 14.3% of men. These results suggest an apparent HL prevalence between sexes, although no statistically significant difference was observed, most likely due to the limited sample size and the imbalance in sex distribution. When comparing these findings with prevalence estimates, the WHO 2020 Hearing Report [31] indicates a global prevalence of moderate or severe HL of approximately 2% among young adults aged 20–29 years, without including data on mild or slight HL, which are the most common forms in this age group and the ones primarily identified in our study. Within this age range and severity, no significant differences between sexes are reported; however, in terms of gender differences overall, global prevalence of moderate or higher levels of hearing loss is slightly higher among males than females, with 217 million males (5.6%) living with hearing loss compared with 211 million females (5.5%). Although no national data are available for the young Spanish population the 2022 CONSTANCES study conducted in France estimated HL prevalence in adults aged 18–25 years at 3.4% in women and 4.4% in men [32]. In that study, HL was defined as a pure-tone average (PTA)—the mean hearing threshold at 500, 1000, and 2000 Hz—in the better ear ≥ 20 dB [33], whereas our study employed the more sensitive ASHA classification, which considers HL from a PTA ≥ 15 dB in the better ear, allowing for earlier detection of subclinical changes. For this reason, the high prevalence observed in our sample is expected.

### 3.2. Relationship Between Antimicrobial Resistance and Auditory Function

In our analysis, young adults with some degree of HL showed higher MICs for nalidixic acid under aerobic conditions, for amoxicillin under anaerobic conditions, and for ciprofloxacin under anaerobic conditions, compared with participants without HL, and these differences reached statistical significance. PCA further revealed a clustering tendency among participants without HL and greater dispersion among those with HL across the antibiotics tested. These findings support the formulation of hypotheses regarding a potential association between antimicrobial resistance patterns and auditory function.

The consistently higher MICs observed in the HL group may reflect cumulative lifetime exposure to antibiotics resulting from infections of diverse origins. Although participants reporting recurrent otitis media were initially excluded from the study, it cannot be ruled out that previous episodes of infection requiring antibiotic treatment could have promoted the selection of less susceptible bacterial strains, thereby contributing to the elevated MIC values observed. Moreover, chronic or repeated exposure to certain antibiotics with potential ototoxic effects might have contributed directly to the onset or progression of HL, suggesting an indirect link between antibiotic exposure, bacterial resistance, and auditory outcomes. This relationship would not be limited to aminoglycosides [34], whose ototoxic potential is well established but unlikely relevant in our cohort given their hospital use. Other antibiotic classes, including macrolides [35], have also been associated with potential auditory toxicity, which may further support this hypothesis. It should be emphasized, however, that individual antibiotic exposure history was not assessed in this study. Therefore, these interpretations remain speculative and should be viewed as exploratory observations that warrant further investigation.

### 3.3. Potential Role of the Intestinal Microbiome

A complementary hypothesis is that chronic or repeated antibiotic exposure may not only promote the selection of resistant bacterial strains but could also potentially alter the intestinal ecosystem in ways reflected not only by changes in microbiome composition but also by enrichment of the gut resistome—the set of antimicrobial resistance genes harbored by gut microbes. An expanded resistome may indicate repeated antibiotic pressures and has been associated with reduced microbial diversity, shifts toward proinflammatory taxa, and decreased ecosystem stability [36,37,38]. The concept of the gut–brain–ear axis [10] provides atheoretical framework for understanding how intestinal dysbiosis, might indirectly influence auditory function. Recent experimental studies in murine models have in fact demonstrated that perturbations in the gut microbiome can modulate systemic inflammation, oxidative stress, and the integrity of physiological barriers such as the blood–brain barrier [39] and the blood–labyrinth barrier within the cochlea, ultimately compromising cochlear homeostasis and the function of sensory hair cells responsible for mechanoelectrical transduction [13]. Although noise exposure was not assessed in our study, it is widely recognized as a potential inducer of both HL and gut microbiome alterations [40]. Animal models indicate that environmental noise may modify microbial composition and metabolic activity, including changes in short-chain fatty acids that regulate neuroinflammation and neuronal function. Communication between the gut and auditory system, mediated through the vagus nerve, sympathetic pathways, and the hypothalamic–pituitary–adrenal axis, might support bidirectional interaction influencing auditory susceptibility [10]. In line with this concept, our analyses revealed that overall gut microbiota composition did not differ markedly between individuals with and without HL; however, the abundance of *Faecalibacterium prausnitzii* was significantly higher in the HL group. This is surprising, as *F. prausnitzii* is typically associated with anti-inflammatory gut environments [41], suggesting a subtle, species-specific shift rather than broad dysbiosis. Similarly, higher resistance to amoxicillin was associated with increased *F. prausnitzii* abundance, whereas no differences were observed for other antibiotics. These observations provide preliminary evidence that specific microbial taxa and resistome features could be linked to auditory status and antibiotic exposure, consistent with the hypothesized gut–microbiome–ear axis. These findings provide a conceptual basis for the gut–microbiome-ear axis; however, the potential contribution of the resistome to these pathways remains largely hypothetical, and further research is necessary to elucidate the underlying mechanisms and causal relationships.

### 3.4. Strengths, Limitations and Future Research

This study represents a pioneering effort to explore the relationship between the intestinal resistome and the auditory function, introducing an innovative approach that integrates microbiome composition, antimicrobial resistance, and auditory health. However, the small sample size and sex imbalance constitute important limitations that reduce the generalizability of the findings and constrain the statistical power to detect subtle differences between groups. Another key limitation is the lack of metagenomic analysis, which restricts the depth of characterization of the gut microbiome and resistome. In addition, key potential confounders, including diet, individual antibiotic use history, and noise exposure, were not fully controlled or quantified, although participants completed a general health and lifestyle questionnaire and individuals with prior use of ototoxic medications or chronic auditory conditions were excluded. Regarding statistical analyses, technical duplicates were performed for each assay; given the small sample size and exploratory nature of this pilot study, formal corrections for multiple testing (e.g., Bonferroni) were not applied. Furthermore, the cross-sectional design precludes the establishment of causal inferences or directionality in the observed associations between antimicrobial resistance, intestinal dysbiosis, and HL. Although causality cannot be inferred, the findings offer a conceptual framework consistent with emerging evidence linking the gut microbiome to auditory physiology. Future longitudinal and experimental studies will be required to determine the temporal and biological direction of these interactions, as well as their potential mechanistic underpinnings. Such studies should also incorporate environmental and lifestyle factors known to influence the microbiome and auditory function, including occupational and recreational noise exposure, dietary patterns, antibiotic use history and sleep quality, to provide a more comprehensive understanding of the gut–microbiome–ear axis and its implications for auditory health [42,43,44,45].

## 4. Materials and Methods

### 4.1. Study Design and Sample Selection

A descriptive, cross-sectional pilot study was conducted in a cohort of 22 healthy young adults aged 18 to 26 years, recruited at CEU San Pablo University (Madrid, Spain). Participant recruitment took place between 25 January and 30 April 2025, within the framework of the global METAEAR project.

Participants were recruited by convenience sampling according to the following eligibility criteria:

- Inclusion criteria: individuals within the specified age range.
- Exclusion criteria: subjects with acute or chronic diseases related to HL, cardiovascular disease, diabetes, and/or cancer; current use or use within the 30 days prior to sample collection of ototoxic drugs, vitamin supplements, probiotics, and/or prebiotics; history of surgical interventions of the auditory system; family history of early-onset HL; or known auditory pathologies such as otitis, otosclerosis, or Ménière’s disease.

All volunteers provided written informed consent prior to participation in the study. The study protocol was approved by the Research Ethics Committee of CEU San Pablo University (approval code: 812/24/116 PRENUT2HEAR), in accordance with the principles of the Declaration of Helsinki.

### 4.2. General Description of the Study Design

Demographic, health, and lifestyle information were collected through a structured screening questionnaire to verify compliance with all inclusion and exclusion criteria. Participants underwent a comprehensive hearing assessment using pure-tone audiometry, and fecal samples were collected for microbiological and antimicrobial resistance analyses.

### 4.3. Sociodemographic, General Health, and Personal Questionnaire

This questionnaire was designed to characterize the demographic and clinical characteristics of the study population. The sociodemographic questionnaire collected detailed information including date and place of birth, nationality, sex, place of residence, and current educational, professional, or employment status. The general health questionnaire gathered data on the participants’ medical history, particularly focusing on auditory disorders such as otitis, otosclerosis, or Ménière’s disease, among others. It also considered diseases associated with HL, including cardiovascular conditions, diabetes, and/or cancer, as well as the use of medications with potential ototoxic effects or vitamin-mineral supplements. This questionnaire additionally served to confirm compliance with the inclusion and exclusion criteria established for participant selection.

### 4.4. Hearing Evaluation

To assess the auditory function, both subjective and objective audiological tests were performed for all volunteers. These assessments were performed by qualified audiology specialists from the Faculty of Pharmacy at CEU San Pablo University, in accordance with Spanish legislation (Royal Decree 1685/2007). The tests assessed the participants’ hearing status and, in cases of HL, determined its origin (conductive, sensorineural, or mixed). All the evaluations were carried out in a soundproof booth and analyzed using the NOAH 4.0 software. Initially, otoscopic examination was performed to examine the outer ear, ear canal, tympanic membrane, and middle ear [46]. This was followed by tuning fork tests (Rinne and Weber) [47] to help differentiate the type of HL. Finally, pure-tone audiometry was conducted to determine both air and bone conduction thresholds using pure tones at frequencies of 125, 250, 500, 750, 1000, 2000, 3000, 4000, and 8000 Hz in 5 dB increments [48].

### 4.5. Gut Microbiome Analysis

Fresh stool samples were collected from each participant and immediately stored at –80 °C until 24 h before processing. Microbiological and antimicrobial resistance analyses were subsequently performed as described below.

To assess antimicrobial resistance in stool samples, bacterial resistance to selected antimicrobials was evaluated using disk diffusion and E-test strips methods to determine the MIC, following the protocol described by Robas Mora et al. [49], under both aerobic and anaerobic conditions. Two batches of ten samples were processed. Sterile culture media were prepared including 0.45% saline solution and Mueller–Hinton agar (Condalab^TM^, Madrid, Spain). Approximately 2 g of each stool sample were mixed with saline under sterile conditions, homogenized using a sonicator, centrifuged, and the resulting supernatant was plated on Mueller–Hinton agar.

For each fecal sample, one biological replicate was prepared, and all antibiotic disk assays were conducted in technical duplicate to ensure reproducibility and reliability. The antibiotics tested included nalidixic acid, amoxicillin, cefotaxime, imipenem (alone and in combination with EDTA), and ciprofloxacin for both aerobic and anaerobic cultures (BioMérieux^TM^, Marcy l’Etoile, France) and were selected based on their known profiles of resistance or sensitivity in representative intestinal microorganisms. These antibiotics have been frequently used in previous studies evaluating antimicrobial resistance in human and animal gut microbiota [50,51,52]. Anaerobic cultures were incubated in an aerobic bag system (Merck KGaA, 64271, Darmstadt, Germany). Disks and E-test strips were placed on the inoculated plates, which were incubated following the manufacturer’s instructions. Plates were incubated at 37 °C according to the manufacturer’s instructions. Inhibition zones were measured at 24 and 48 h for aerobic cultures and at 24 h for anaerobic cultures to assess MIC values.

For the qPCR analysis, total DNA was extracted from 22 fecal samples using the NucleoSpin^TM^ Soil DNA Kit (Macherey-Nagel, GmbH & Co., Düren, Germany) according to the manufacturer’s instructions and eluted in a final volume of 100 µL. The characterization of gut bacterial species was performed at GoodGut S.L.U. (Girona, Spain) by quantifying 15 microbial markers representing the main phyla, genera, and groups of the gut microbiota: *Akkermansia muciniphila*, Bacteroidota, *Candida albicans*, *Clostridium* cluster I, *Escherichia coli*, *Enterococcus* sp., *Faecalibacterium prausnitzii*, Bacillota (Firmicutes), Gammaproteobacteria, *Lactobacillus* sp., *Methanobrevibacter smithii*, *Roseburia* sp., *Ruminococcus* spp., *Clostridium* cluster XIV, and Eubacteria [53].

Reactions for each marker were performed individually using GoTaq^®^ qPCR Bryt Master Mix (Promega Corporation, Madison, WI, USA) or GoTaq^®^ qPCR Probe Master Mix (Promega Corporation, Madison, WI, USA), depending on the target. Each reaction had a final volume of 10 µL, containing 12–20 ng of genomic DNA. Primers and probes were obtained from Macrogen Inc., Seoul, South Korea. All samples were analyzed in duplicate alongside negative controls and standard curves using an AriaDx thermocycler (Agilent Technologies, Santa Clara, CA, USA), in compliance with ISO 13485 standards [53].

### 4.6. Statistical Analysis

Data were expressed as median and interquartile range for quantitative variables, or as frequencies for qualitative variables. Normality was checked using the non-parametric Kolmogorov–Smirnov test. Given the non-normal distribution of the data, the non-parametric Mann–Whitney U test was applied to evaluate differences between groups. For comparative analysis of microbiome resistance patterns, data derived from antibiotic resistance were integrated and dimensionally reduced into two principal components explaining most of the model variability. PCA was then performed, and the resulting components were plotted in a two-dimensional space according to hearing status (presence or absence of HL) to visualize clustering tendencies and potential group differentiation.

All statistical analyses were performed using IBM SPSS Statistics v29.0 (IBM Corp., Armonk, NY, USA).

## 5. Conclusions

In this cohort of young adults, a notable prevalence of mild or slight HL was observed, particularly among women. The results demonstrated a significant association between HL and higher MICs for nalidixic acid, amoxicillin, and ciprofloxacin, together with a trend toward increased MIC values and greater variability in the HL group for other antibiotics such as cefotaxime and imipenem. qPCR analyses of gut microbiota revealed that the abundance of *Faecalibacterium prausnitzii* was significantly higher in individuals with HL, and this species was also enriched in participants exhibiting greater resistance to amoxicillin.

These findings suggest that specific microbial taxa and resistome features may be linked to both auditory status and antibiotic exposure, reinforcing the emerging concept of a gut–ear–brain axis. They underscore the importance of monitoring gut microbiome integrity and cumulative antibiotic exposure in young and potentially susceptible populations. Moreover, the observed associations provide a rationale for future longitudinal and mechanistic studies aimed at exploring microbiome-targeted strategies to preserve auditory health and mitigate the potential impact of antibiotic-induced microbiome perturbations.

## Figures and Tables

**Table 1:** Distribution of participants by sex and hearing loss category.

	Women	Men	Total
No HL	7 (46.7%)	6 (85.7%)	13 (59.1%)
Any degree of HL	8 (53.3%)	1 (14.3%)	9 (40.9%)

HL: hearing loss.

## Data Availability

The data presented in this study are available on request from the corresponding author.

## Abbreviations

The following abbreviations are used in this manuscript:

ASHA	American Speech-Language-Hearing Association
AMR	Antimicrobial Resistance
HPA	Hypothalamic–pituitary–adrenal axis
HL	Hearing loss
MIC	Minimum inhibitory concentration
PCA	Principal component analysis
PLDs	Personal Listening Devices
PTA	Pure Tone Average
qPCR	Quantitative polymerase chain reaction
WHO	World Health Organization
